# Differences between hard and soft phylogenetic data

**DOI:** 10.1098/rspb.2017.2150

**Published:** 2017-12-13

**Authors:** Robert S. Sansom, Matthew A. Wills

**Affiliations:** 1 School of Earth and Environmental Sciences, University of Manchester, Manchester M13 9PT, UK; 2 Milner Centre for Evolution, Department of Biology and Biochemistry, University of Bath, Bath BA2 7AY, UK

**Keywords:** phylogenetics, morphology, partition, heterogeneity, missing data

## Abstract

When building the tree of life, variability of phylogenetic signal is often accounted for by partitioning gene sequences and testing for differences. The same considerations, however, are rarely applied to morphological data, potentially undermining its use in evolutionary contexts. Here, we apply partition heterogeneity tests to 59 animal datasets to demonstrate that significant differences exist between the phylogenetic signal conveyed by ‘hard’ and ‘soft’ characters (bones, teeth and shells versus myology, integument etc). Furthermore, the morphological partitions differ significantly in their consistency relative to independent molecular trees. The observed morphological differences correspond with missing data biases, and as such their existence presents a problem not only for phylogeny reconstruction, but also for interpretations of fossil data. Evolutionary inferences drawn from clades in which hard, readily fossilizable characters are relatively less consistent and different from other morphology (mammals, bivalves) may be less secure. More secure inferences might be drawn from the fossil record of clades that exhibit fewer differences, or exhibit more consistent hard characters (fishes, birds). In all cases, it will be necessary to consider the impact of missing data on empirical data, and the differences that exist between morphological modules.

## 1. Introduction

Phylogenetic trees are vital for inferring patterns and rates of evolutionary change and for testing evolutionary hypotheses. When building trees using genomic data, heterogeneities of signals and rates are acknowledged and taken into account by applying different models to partitions of genes and sites [1,2]. This has become more important as molecular datasets have grown in size from single loci to genome scale, and the range and sophistication of models has increased. Morphological systematists, by contrast, have historically analysed a broad diversity of morphological characters holistically and there is no tradition of testing for homogeneity or of partitioning analyses. However, there is growing evidence that some aspects of morphology or classes of characters are more phylogenetically informative than others, and similarly that homoplasy is not homogenously distributed [3–5]. That differences between classes of morphological data exist is problematic in and of itself, and those differences need to be recognized and accounted for. It is especially problematic in instances where those differences correspond with missing data biases; if only some selected subset of data is available for phylogenetic inference, and that subset is not representative of the complete phylogenetic data as a whole, then the resultant trees will be systematically distorted.

Simulation studies have indicated that missing data need not obfuscate the inference of accurate phylogeny *per se*; the quality of the data that remains is far more important than the quantity of data lost [6–10] (although [11,12]). Characters within a simulated dataset are often derived from a single underlying model; as such, data filters are effectively random even when missing data are concentrated in taxa and characters because signal is homogenously distributed across characters. Empirical data biases, however, are far from random. Missing entries are concentrated within particular predictable classes of characters, and phylogenetic differences have been demonstrated between readily fossilizable and less fossilizable classes of characters (e.g. dental versus skeletal characters [5] and cranial versus postcranial characters [3]). The most fundamental fossilization bias is the loss of soft tissues. Muscles and nerves are rarely preserved in fossils [13], while biomineralized hard tissues such as bones, teeth and shells are much more prevalent. The differences in phylogenetic signal conveyed by ‘hard’ and ‘soft’ morphological characters have yet to be systematically investigated. Meta-analysis of data from modern clades [4] has demonstrated that soft-part characters make a relatively disproportionate contribution towards recovering nodes in the phylogeny, while their omission also resulted in the phenomenon of ‘stemward slippage’ whereby taxa resolve erroneously closer to the root of the tree [4,14,15]. In more limited studies of individual clades, the picture is more nuanced. For example, qualitative and variously quantitative investigations of morphological data in gastropods disagree as to which class of characters is more informative [16–20]. In hominoids, soft-part characters have been found to be more congruent than osteological characters when optimized onto independent molecular trees [21]. Similarly, in toothcarp fishes [22] and amniotes [23], the characters from soft tissues are essential for obtaining a resolved phylogeny that recovers established higher taxa. In a study of catfish [24], the distributions of autapomorphies were used to infer differences between hard and soft characters. While these examples demonstrate that the signals in hard and soft characters can differ markedly, it also highlights the disparity of methods employed to quantify those differences. Furthermore, it demonstrates that the patterns may vary across the tree of life. As such, systematic application of a unified set of methods across a broad range of taxa is necessary.

Here, we test for differences in the phylogenetic signal conveyed by ‘hard’ and ‘soft’ morphological characters across 59 datasets of extant clades. Firstly, we test for partition homogeneity using the incongruence length difference (ILD) test [25,26] and the incongruence relationship difference test (IRD) [3]. Secondly, we test for differences between the two classes of characters in terms of compatibility with independent molecular trees. Thirdly, we test whether any differences are distributed evenly or whether they are concentrated in particular animal clades. These combined analyses constitute the first systematic and taxonomically inclusive exploration of differences in phylogenetic signal between fossilizable and less-fossilizable morphological characters. By systematically addressing these questions in a disparate and wide-ranging sample of groups at different taxonomic levels, our conclusions will be relevant to a large breadth of the metazoan fossil record rather than just a few limited case studies.

## 2. Methods

### (a) Partition homogeneity

**Figure 1.**
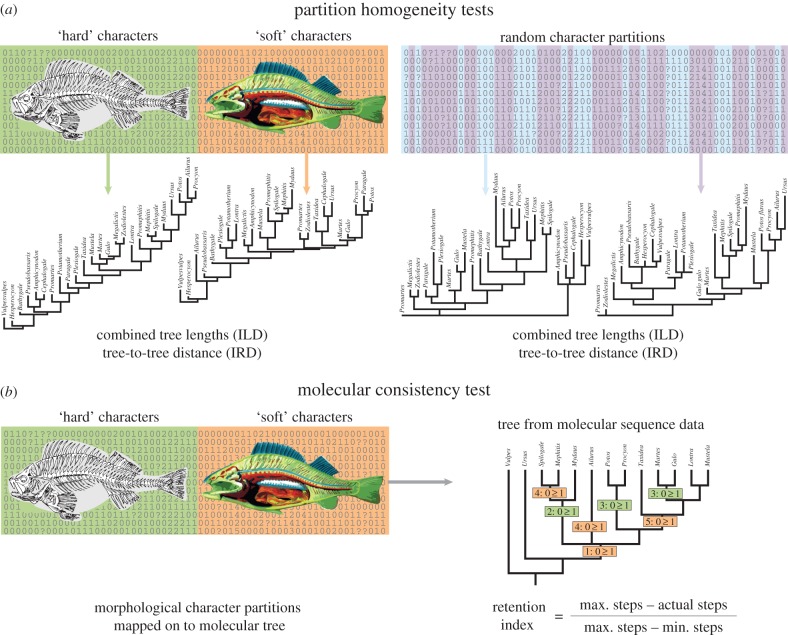
Tests applied to morphological character partitions. Partition homogeneity tested (*a*) by the Incongruence Length Difference (ILD) test and Incongruence Relationship Difference (IRD) test; the trees resulting from searches using each partition are compared with trees resulting from searches using random partitions of the same size in terms of tree length summed for the two partitions or average nearest neighbour tree-to-tree distance between most parsimonious trees of the two partitions. Molecular consistency tested (*b*) by optimizing morphological data onto molecular trees and comparing the resulting retention indices of characters and partitions. (Online version in colour.)

Morphological datasets for extant clades comprising both hard and soft character partitions were previously identified and edited by Sansom & Wills [4]. Nine of the original 79 datasets were omitted because of their taxonomic overlap with others in the sample. All datasets have a minimum of 10 taxa and a minimum 20% of characters from either partition [4]. The null hypothesis that hard and soft partitions convey a homogenous phylogenetic signal was tested using the ILD test [26] and the incongruence relationship distance (IRD) test [3]. For both, ordering of characters was applied as specified in the original studies. In the ILD test, each matrix partition is subjected to independent parsimony analyses, which each yield lengths of the optimal trees, which are then summed (figure 1). The characters are then randomly partitioned repeatedly (999 times in this case) using the same proportions as the original. If the summed lengths of the trees from the original partitions falls outside the range of the combined lengths of trees from the randomized partitions (e.g. 5% of extremes of range), then the signals within the original partitions are deemed to be significantly incongruent (*p* < 0.05). Criticisms of the ILD test have centred on its high type I error rate (false positives); this may be a function of the nonlinear response of the ILD to asymmetries in the distribution of noise [27], along with reporting bias and a tendency to test partitions already suspected of incongruence [3]. The IRD test is analogous to the ILD in that it compares the original character partitions with repeatedly randomized partitions of equivalent size, but differs in that it uses tree-to-tree metrics rather than sums of tree lengths. For the IRD, each most parsimonious tree resulting from searches using one partition was compared with all the most parsimonious trees resulting from searches using the other partition to derive Robinson–Foulds distances [28]. The mean distance between nearest neighbours in the two sets of most parsimonious trees is used as a measure of incongruence [3,5]. TNT scripts [29] were used for each.

### (b) Molecular consistency

Length- and relationship-based partition tests provide useful indices of incongruence between hard and soft characters *within* a matrix. However, in order to test whether one matrix partition contains a signal that more accurately reflects evolutionary history, it becomes necessary to compare morphological data with some independent source of phylogenetic information. In this case, we have used molecular trees to calculate the relative levels of homoplasy. Comparisons of molecular and morphological data have demonstrated incongruence between the data classes [30]; here partitions of morphological data are investigated to determine whether they exhibit differing levels of congruence with molecular data. Molecular trees for each dataset were sourced from the literature (following [5]). Priority was given to the molecular tree with the leaf set that overlapped most strongly with that in the morphological dataset; the underlying amount of sequence data was used as a secondary criterion (see the electronic supplementary material). Datasets for which adequate molecular data coverage was not available (i.e. a minimum of 10 taxa) were excluded (see the electronic supplementary material). Morphological characters were optimized onto molecular trees and retention indices [31] used as a measure of homoplasy both for individual characters (ri) and for individual partitions (RI). Absolute values of the ensemble retention index (RI) are directly related to the number of taxa, number of characters and distribution of homoplasy within the dataset in question. Here however, retention indices for morphological characters and partitions are derived through application to independent molecular trees rather than to trees inferred from those same morphological characters. Furthermore, comparisons of retention indices are made within datasets. As such, the number of taxa and characters is eliminated as conflating factors and relative RIs provide a direct test of distributions of homoplasy across partitions. Taxa not present in the molecular trees were excluded from calculations of character homoplasy. Characters that were not informative (autapomorphic or invariant) for the subset of taxa present in the molecular tree were also omitted from calculations. Differences between hard and soft partitions were tested within individual datasets (Mann–Whitney–Wilcoxon (MWW) of individual character retention indices) and across all datasets (ANOVAs of retention indices of partitions with repeated measures for datasets). The sources of the molecular trees are given in the electronic supplementary material.

## 3. Results

‘Hard’ and ‘soft’ morphological character partitions were analysed in 59 datasets (electronic supplementary material) representing a total of 2478 taxa and 9681 characters. Partition homogeneity tests found significant differences in the signal conveyed by the hard and soft partitions within datasets. Twenty-one of 59 datasets were significant (*p* < 0.05) for the ILD test, while 14 of 57 were significant (*p* < 0.05) for the IRD test. Combining *p*-values across all datasets (Fisher's combined probability) finds high significance (*p* = 5.5 × 10^−22^ for ILD and *p* = 2.8 × 10^−10^ for IRD). Tests for differences in molecular consistency also found significant differences between the hard and soft morphological character partitions. Retention indices of characters in each partition mapped onto molecular trees were found to be significantly different within datasets for 19 of the 59 datasets (*p* < 0.05 for MWW). Combining *p*-values across all datasets again yielded highly significant differences (*p* = 4.7 × 10^−22^ for Fisher's combined probability).

**Figure 2.**
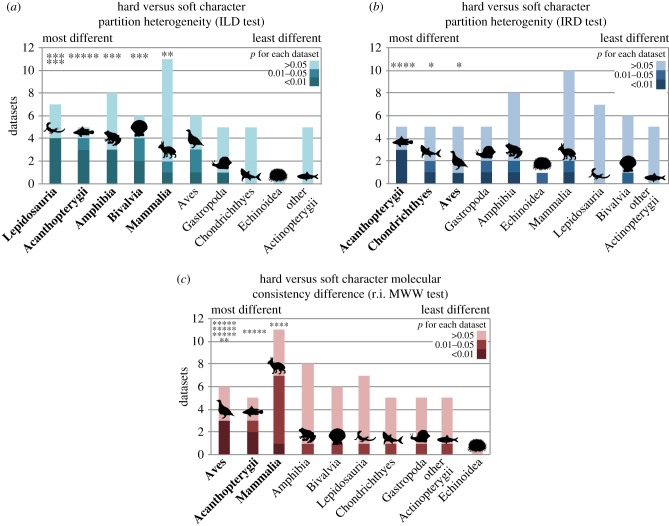
Results of partition tests. Datasets are grouped by class and are colour coded according to their individual *p*-value for (*a*) the Incongruence Length Difference (ILD) test, (*b*) the Incongruence Relationship Difference (IRD) test, and (*c*) the molecular consistency difference test (i.e. MWW test of retention indices of characters in each partition relative to molecular trees). The grouped classes are arranged from those exhibiting most differences (left) to least differences (right) (Fisher's combined probability test derived from combined *p*-values of datasets in each clade). Classes with combined significance are in bold and highlighted with asterisks denoting the level of significance. (Online version in colour.)

The 59 datasets represent nine different vertebrate and invertebrate classes: Bivalvia (*n* = 6), Gastropoda (*n* = 5), Echinoidea (*n* = 1), Chondrichthyes (*n* = 5), Actinopterygii (*n* = 10), Amphibia (*n* = 8), Lepidosauria (*n* = 7), Aves (*n* = 6) and Mammalia (*n* = 11). Although turtles (*n* = 1) have been placed as sister taxa to archosaurs [32], they are grouped with lepidosaur reptiles here for gross morphological reasons. The distribution of the observed differences between partitions varies substantially across clades (figure 2). Combining *p*-values of the ILD test across datasets of each class finds significant partition heterogeneity in Lepidosauria, Actinopterygii, Amphibia, Bivalvia and Mammalia (Fisher's method *p* = 4.5 × 10^−8^, 3.8 × 10^−5^, 2.7 × 10^−5^, 4.0 × 10^−5^ and 4.5 × 10^−4^, respectively, all significant at *p* < 0.0056 when correcting for multiple comparisons). For the IRD test, combined partition homogeneity is significant for Actinopterygii, Chondrichthyes and Aves (Fisher's combined probability *p* = 4.1 × 10^−5^, 0.0020 and 0.0038, respectively). Combining the results of the tests for partition molecular consistency differences (MWW of retention indices) found significant differences in Aves, Actinopterygii and Mammalia (Fisher's combined probability *p* = 3.3 × 10^−19^, 2.7 × 10^−6^, 3.1 × 10^−6^, respectively). Given the marked difference observed within the Actinopterygii, they were split into two groups, the Acanthopterygii (*n* = 5) and non-acanthopterygian Actinopterygii (*n* = 5) (figure 2).

**Figure 3.**
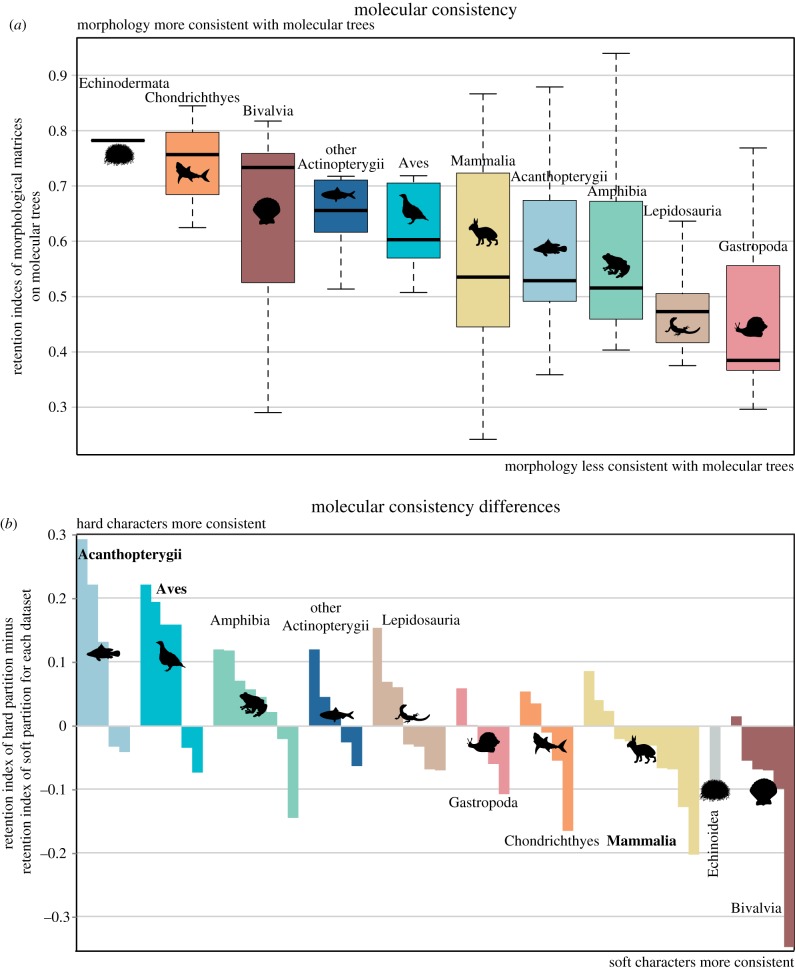
Molecular consistency of datasets for each class. The absolute molecular consistency values (*a*) are arranged in order of average RI for each class (left being highest consistency between morphology and molecules, right being lowest). The molecular consistency differences (*b*) for each dataset are arranged by average direction of difference for each class (position of cartoons); on the left are datasets for which the hard characters are more consistent with molecular trees relative to soft characters, while those on the right are datasets for which the hard characters are less consistent with molecular trees relative to soft characters. Those clades for which significant differences were found in the molecular consistency tests (figure 2*c*) are in bold. (Online version in colour.)

The classes exhibited different levels of morphological consistency with molecular trees (figure 3*a*); Chondrichthyes and Bivalvia exhibit high compatibility between morphological matrices and molecular trees (average RI > 0.7) while Lepidosauria and Gastropoda exhibit low compatibility (average RI < 0.5). Nevertheless, the variability of molecular consistency between classes is not significant (one-way ANOVA, *p* = 0.164). Plotting the direction of differences between hard and soft partitions in terms of molecular consistency also shows variation across clades (figure 3). This variability is significant (mixed ANOVA of partition type and clade with repeated measures for datasets, gives *p* = 0.010 for clade). Acanthopterygii and Aves both showed relatively higher retention indices for hard characters optimized onto molecular trees, while Mammalia and Bivalvia showed relatively higher retention indices for soft characters optimized onto molecular trees. Echinoidea also exhibit relatively low consistency of hard characters with molecular trees, but this is based on one morphological dataset. The other classes were generally more mixed.

## 4. Discussion

Differences between the phylogenetic signal carried by hard-part and soft-part characters were found to be a widespread problem in data matrices from a wide range of animal groups. Not only did partition tests find significant differences between the phylogenetic signal conveyed by these partitions, but those partitions were also found to differ in their consistency relative to independent molecular trees. These differences potentially undermine our ability to use palaeontological data to test evolutionary hypotheses. In order to determine the magnitude of this problem, it is necessary to look at the distribution of significant results across taxa (figure 3). The ILD test found significant differences in reptiles, spiny-finned fishes, amphibians, bivalves and mammals while the IRD test found significant differences in spiny-finned fishes, cartilaginous fishes and birds. The molecular consistency tests found significant differences in birds, spiny-finned fishes and mammals. Although the tests give different insights [3], certain groups (Acanthopterygii, Aves and Mammalia) consistently exhibited differences between hard-part and soft-part characters across the different tests applied. The implications of these observed differences depend on whether we can interpret either partition as better representing actual evolutionary history. For spiny-finned fish and birds, the more readily fossilizable characters (osteology) are more consistent with independent benchmarks (i.e. molecular trees). As such, phylogenetic and evolutionary inferences drawn from fossil spiny-finned fish and birds (including dinosaurs as part of the avian stem) may be secure. On the other hand, mammals and bivalves exhibit significant differences, and it is their more readily fossilizable characters (osteology and conchology) that are less consistent with molecular trees. As such, evolutionary inferences drawn from fossil mammals and bivalves may be less secure. This potentially compounds the differences already observed between dental and osteological characters in mammals coupled with the enhanced preservation potential of teeth [5]. Some clades exhibit few significant differences between partitions and little evidence of directional differences, namely other fishes, chondrichthyans, gastropods and reptiles. As such, we might infer that inferences drawn from these clades are secure as phylogenetic signal is apparently homogenously distributed across morphological partitions. However, for both gastropods and reptiles, the ‘base-line’ of consistency between all morphology and molecular trees is low (average retention indices less than 0.5, figure 3*a*) which could potentially undermine evolutionary inferences drawn from morphology.

What underlies these observed differences between hard and soft characters? Different rates of character evolution in modules (functionally and developmentally integrated suites) could result in different levels of homoplasy in morphological partitions. This is unlikely in certain cases here; for example, osteology and myology are intrinsically linked and integrated systems that might be expected to evolve in concert. However, the majority of datasets that exhibited significant differences comprised characters from less closely related modules, for example osteology and integument (colour patterns and structure of skin, scales or feathers) or conchology and internal anatomy. Alternatively, it is possible that the differences do not result from evolutionary processes, but a biased or insufficient sampling of characters for one of the two partitions. Molecular compatibility tests are unable to distinguish between modularity and poor sampling; either phenomenon could result in elevated homoplasy (and lower retention indices) of the characters in one partition. Nevertheless, the taxonomic clustering of significant differences occurs in (i) diverse clades, (ii) data matrices produced by a wide-range of unrelated researchers, and (iii) ostensibly loosely connected modules. Taken together those observations suggest genuine differences exist between hard and soft characters. Furthermore, the numerous data matrices tested are not only the best available data set to test these hypotheses, but also the most suitable for integrating fossils into phylogenies of predominately extant taxa.

## 5. Conclusion

The differences observed here between hard and soft characters present a real and considerable problem for reliably inferring the phylogenetic relationships of particular clades. These sorts of differences will need accounting for in phylogenetic analyses of extant taxa, but especially in analyses of extinct taxa. Detailed comparisons of the morphological and molecular data for extant representatives of the clades in question may ameliorate this problem; meta-analyses may enable identification of reliable character suites within fossilizable data, and could enable tests to distinguish between modularity and poor sampling as causes of the observed incongruence. Without this additional data, it may be necessary to apply caution to phylogenetic analysis of extinct taxa in the clades identified as problematic above, notably mammals and bivalves. However, no systematic differences between hard and soft-part characters were observed in chondrichthyans and other non-acanthopterygian fishes. Moreover, in acanthopterygian fishes and birds, preservable characters are more consistent with molecular data than less preservable ones. We therefore recommend that the distinction between clades with and without poor taphonomic fidelity is taken into account when choosing model systems for large-scale macro-analyses; non-mammalian vertebrates may provide better model systems than mammals and bivalves. Our results also serve as a valuable reminder that palaeontologists need to carefully consider the impact of missing data on their analyses.

## Supplementary Material

Supplementary Information

## Data accessibility

Data are available from the Dryad Digital Repository (https://doi.org/10.5061/dryad.541pt) [33].
